# 4-{[(*E*)-(3-Phenyl-1*H*-pyrazol-4-yl)methyl­idene]amino}-1*H*-1,2,4-triazole-5(4*H*)-thione

**DOI:** 10.1107/S1600536811024834

**Published:** 2011-07-09

**Authors:** Hoong-Kun Fun, Madhukar Hemamalini, Shridhar Malladi, Arun M. Isloor

**Affiliations:** a X-ray Crystallography Unit, School of Physics, Universiti Sains Malaysia, 11800 USM, Penang, Malaysia; b Department of Chemistry, National Institute of Technology, Karnataka, Surathkal, Mangalore 575 025, India

## Abstract

In the title compound, C_12_H_10_N_6_S, a weak intra­molecular C—H⋯S hydrogen bond stabilizes the mol­ecular conformation. The pyrazole and triazole rings form a dihedral angle of 17.82 (8)°. The mol­ecule adopts an *E* configuration with respect to the central C=N double bond. In the crystal, inter­molecular N—H⋯N and N—H⋯S hydrogen bonds link mol­ecules into chains propagating in [20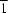].

## Related literature

For applications of Schiff bases, see: Kahveci *et al.* (2005 ▶); Bekircan *et al.* (2006 ▶); Singh & Dash (1988 ▶). For a related structure, see: Fun *et al.* (2010 ▶).
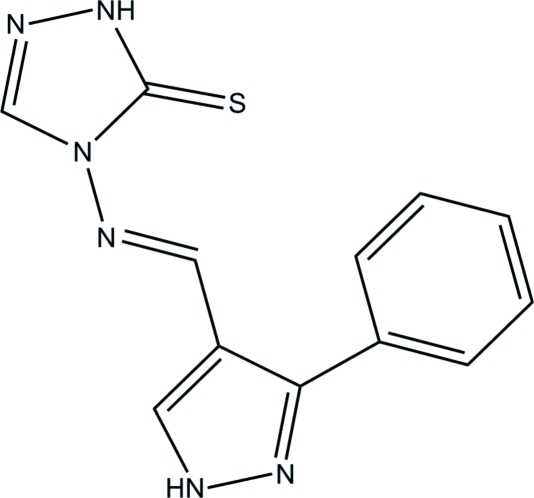


## Experimental

### 

#### Crystal data

C_12_H_10_N_6_S
*M* *_r_* = 270.32
Monoclinic, 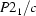

*a* = 4.1180 (4) Å
*b* = 17.9237 (16) Å
*c* = 17.0787 (15) Å
β = 97.352 (3)°
*V* = 1250.2 (2) Å^3^
*Z* = 4
Mo *K*α radiation
μ = 0.25 mm^−1^
*T* = 296 K
0.55 × 0.26 × 0.19 mm

#### Data collection

Bruker APEXII DUO CCD area-detector diffractometer
Absorption correction: multi-scan (*SADABS*; Bruker, 2009 ▶) *T*_min_ = 0.872, *T*_max_ = 0.954
14262 measured reflections
4361 independent reflections
3204 reflections with *I* > 2σ(*I*)
*R*_int_ = 0.022

#### Refinement

*R*[*F* ^2^ > 2σ(*F* ^2^)] = 0.040
*wR*(*F* ^2^) = 0.120
*S* = 1.03
4361 reflections
180 parameters
H atoms treated by a mixture of independent and constrained refinement
Δρ_max_ = 0.29 e Å^−3^
Δρ_min_ = −0.28 e Å^−3^

### 

Data collection: *APEX2* (Bruker, 2009 ▶); cell refinement: *SAINT* (Bruker, 2009 ▶); data reduction: *SAINT*; program(s) used to solve structure: *SHELXTL* (Sheldrick, 2008 ▶); program(s) used to refine structure: *SHELXTL*; molecular graphics: *SHELXTL*; software used to prepare material for publication: *SHELXTL* and *PLATON* (Spek, 2009 ▶).

## Supplementary Material

Crystal structure: contains datablock(s) global, I. DOI: 10.1107/S1600536811024834/cv5118sup1.cif

Structure factors: contains datablock(s) I. DOI: 10.1107/S1600536811024834/cv5118Isup2.hkl

Supplementary material file. DOI: 10.1107/S1600536811024834/cv5118Isup3.cml

Additional supplementary materials:  crystallographic information; 3D view; checkCIF report

## Figures and Tables

**Table 1:** Hydrogen-bond geometry (Å, °)

*D*—H⋯*A*	*D*—H	H⋯*A*	*D*⋯*A*	*D*—H⋯*A*
N2—H1*N*2⋯S1^i^	0.84 (2)	2.59 (2)	3.3593 (15)	153.6 (17)
N6—H1*N*6⋯N1^ii^	0.90 (2)	1.91 (2)	2.7884 (15)	168.0 (2)
C10—H10*A*⋯S1	0.93	2.50	3.2183 (13)	134

Symmetry codes: (i) 
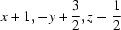
; (ii) 
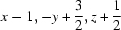
.

**Fig. 1.**
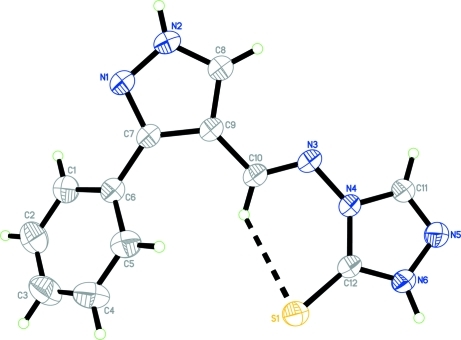
The molecular structure of (I) showing 30% probability displacement ellipsoids and the atom-numbering scheme. Dashed line denotes weak intramolecular hydrogen bond.

## supplementary crystallographic information

### Comment

Schiff bases derived from various heterocycles have been reported to possess antimicrobial (Kahveci *et al.*, 2005), anticancer (Bekircan *et al.*, 2006) and antifungal (Singh *et al.*, 1988) activities. Recently, we have reported the crystal structure of 3-Ethyl-6-[3-(4-fluorophenyl)-1*H*-pyrazole-4-yl]- 1,2,4-triazolo[3,4-*b*][1,3,4]thiadiazole (Fun *et al.*, 2010). In continuation of our studies of triazole derivatives, the title compound, (I), has been synthesized. Herewith we present its crystal structure.

In (I) (Fig. 1), the central pyrazole ring  (N1–N2/C7–C9) makes dihedral angles of 37.64 (8) and 17.82 (8)° with the adjacent phenyl (C1–C6) and triazole (N4–N6/C11–C12) rings, respectively. The dihedral angle between the terminal phenyl (C1–C6) and triazole (N4–N6/C11–C12) rings is 47.08 (9)°.  The molecule adopts an *E* configuration about the central C10═N3  double bond.

In the crystal structure, intermolecular N6—H1N6···N1 and N2—H1N2···S1 (Table 1) hydrogen bonds link the molecules into chains propagated in [2 0 -1].

### Experimental

An equimolar mixture of 4-amino-4*H*-1,2,4-triazole-3-thiol (0.116 g, 0.001 mol) and 3-phenyl-1*H*-pyrazole-4-carbaldehyde (0.172 g, 0.001) in ethanol were refluxed for 7–8 hours in presence of catalytic amount of sulfuric acid. The precipitated solid was filtered, washed with ethanol and recrystallised from ethanol-dioxane mixture. Yield: 0.214 g, 79.25%. M.p- 526–528 K.

### Refinement

Atoms H1N2 and H1N6 were located from a difference Fourier maps and isotropically refined. The remaining H atoms were positioned geometrically [C–H = 0.93 Å] and were refined using a riding model, with *U*_iso_(H) = 1.2 *U*_eq_(C).

### Crystal data

**Table:**

C_12_H_10_N_6_S	*F*(000) = 560
*M**_r_* = 270.32	*D*_x_ = 1.436 Mg m^−^^3^
Monoclinic, *P*2_1_/*c*	Mo *K*α radiation, λ = 0.71073 Å
Hall symbol: -P 2ybc	Cell parameters from 4488 reflections
*a* = 4.1180 (4) Å	θ = 2.7–32.1°
*b* = 17.9237 (16) Å	µ = 0.25 mm^−^^1^
*c* = 17.0787 (15) Å	*T* = 296 K
β = 97.352 (3)°	Block, colourless
*V* = 1250.2 (2) Å^3^	0.55 × 0.26 × 0.19 mm
*Z* = 4	

### Data collection

**Table:**

Bruker APEXII DUO CCD area-detector diffractometer	4361 independent reflections
Radiation source: fine-focus sealed tube	3204 reflections with *I* > 2σ(*I*)
graphite	*R*_int_ = 0.022
φ and ω scans	θ_max_ = 32.2°, θ_min_ = 1.7°
Absorption correction: multi-scan (*SADABS*; Bruker, 2009)	*h* = −5→6
*T*_min_ = 0.872, *T*_max_ = 0.954	*k* = −23→26
14262 measured reflections	*l* = −24→25

### Refinement

**Table:**

Refinement on *F*^2^	Primary atom site location: structure-invariant direct methods
Least-squares matrix: full	Secondary atom site location: difference Fourier map
*R*[*F*^2^ > 2σ(*F*^2^)] = 0.040	Hydrogen site location: inferred from neighbouring sites
*wR*(*F*^2^) = 0.120	H atoms treated by a mixture of independent and constrained refinement
*S* = 1.03	*w* = 1/[σ^2^(*F*_o_^2^) + (0.0574*P*)^2^ + 0.2417*P*] where *P* = (*F*_o_^2^ + 2*F*_c_^2^)/3
4361 reflections	(Δ/σ)_max_ = 0.001
180 parameters	Δρ_max_ = 0.29 e Å^−^^3^
0 restraints	Δρ_min_ = −0.28 e Å^−^^3^

### Special details

**Table:**

Geometry. All s.u.'s (except the s.u. in the dihedral angle between two l.s. planes) are estimated using the full covariance matrix. The cell s.u.'s are taken into account individually in the estimation of s.u.'s in distances, angles and torsion angles; correlations between s.u.'s in cell parameters are only used when they are defined by crystal symmetry. An approximate (isotropic) treatment of cell s.u.'s is used for estimating s.u.'s involving l.s. planes.
Refinement. Refinement of F^2^ against ALL reflections. The weighted R-factor wR and goodness of fit S are based on F^2^, conventional R-factors R are based on F, with F set to zero for negative F^2^. The threshold expression of F^2^ > 2σ(F^2^) is used only for calculating R-factors(gt) etc. and is not relevant to the choice of reflections for refinement. R-factors based on F^2^ are statistically about twice as large as those based on F, and R- factors based on ALL data will be even larger.

### Fractional atomic coordinates and isotropic or equivalent isotropic displacement parameters (Å^2^)

**Table:**

	*x*	*y*	*z*	*U*_iso_*/*U*_eq_	
S1	0.40558 (10)	0.78578 (2)	0.54047 (2)	0.04665 (12)	
N1	0.9965 (3)	0.79730 (6)	0.20882 (6)	0.0407 (3)	
N2	1.0317 (3)	0.72254 (7)	0.20392 (7)	0.0420 (3)	
N3	0.5720 (3)	0.66043 (6)	0.39821 (6)	0.0384 (2)	
N4	0.3925 (3)	0.64999 (5)	0.46129 (6)	0.0348 (2)	
N5	0.1337 (4)	0.57751 (7)	0.53767 (7)	0.0488 (3)	
N6	0.1665 (3)	0.64885 (6)	0.56639 (7)	0.0413 (3)	
C1	0.6630 (4)	0.93695 (8)	0.23797 (9)	0.0472 (3)	
H1A	0.6471	0.9232	0.1851	0.057*	
C2	0.5789 (5)	1.00854 (9)	0.25766 (11)	0.0598 (4)	
H2A	0.5067	1.0426	0.2181	0.072*	
C3	0.6017 (5)	1.02944 (9)	0.33542 (12)	0.0641 (5)	
H3A	0.5426	1.0774	0.3487	0.077*	
C4	0.7125 (5)	0.97907 (10)	0.39391 (11)	0.0629 (5)	
H4A	0.7289	0.9935	0.4466	0.075*	
C5	0.7998 (4)	0.90706 (9)	0.37484 (9)	0.0491 (3)	
H5A	0.8769	0.8736	0.4145	0.059*	
C6	0.7714 (3)	0.88523 (7)	0.29628 (7)	0.0370 (3)	
C7	0.8527 (3)	0.80877 (7)	0.27359 (7)	0.0341 (2)	
C8	0.9156 (3)	0.68630 (7)	0.26224 (8)	0.0388 (3)	
H8A	0.9153	0.6349	0.2698	0.047*	
C9	0.7949 (3)	0.73971 (7)	0.30963 (7)	0.0337 (2)	
C10	0.6185 (3)	0.72707 (7)	0.37639 (7)	0.0358 (2)	
H10A	0.5400	0.7671	0.4030	0.043*	
C11	0.2728 (4)	0.58040 (7)	0.47418 (8)	0.0450 (3)	
H11A	0.2897	0.5399	0.4410	0.054*	
C12	0.3230 (3)	0.69554 (7)	0.52237 (7)	0.0335 (2)	
H1N2	1.114 (5)	0.7046 (11)	0.1654 (12)	0.064 (6)*	
H1N6	0.095 (5)	0.6601 (12)	0.6124 (12)	0.067 (6)*	

### Atomic displacement parameters (Å^2^)

**Table:**

	*U*^11^	*U*^22^	*U*^33^	*U*^12^	*U*^13^	*U*^23^
S1	0.0665 (2)	0.03563 (18)	0.04225 (19)	−0.01109 (15)	0.02386 (16)	−0.00666 (13)
N1	0.0537 (7)	0.0369 (6)	0.0350 (5)	−0.0016 (4)	0.0193 (5)	0.0000 (4)
N2	0.0546 (7)	0.0397 (6)	0.0357 (5)	0.0009 (5)	0.0210 (5)	−0.0030 (4)
N3	0.0492 (6)	0.0373 (5)	0.0323 (5)	0.0024 (4)	0.0192 (4)	0.0020 (4)
N4	0.0459 (6)	0.0294 (5)	0.0322 (5)	0.0016 (4)	0.0167 (4)	0.0020 (4)
N5	0.0741 (8)	0.0330 (6)	0.0443 (6)	−0.0067 (5)	0.0264 (6)	−0.0002 (4)
N6	0.0590 (7)	0.0339 (5)	0.0354 (5)	−0.0037 (5)	0.0225 (5)	−0.0012 (4)
C1	0.0609 (9)	0.0393 (7)	0.0429 (7)	0.0019 (6)	0.0128 (6)	0.0037 (5)
C2	0.0743 (11)	0.0391 (8)	0.0677 (10)	0.0064 (7)	0.0150 (9)	0.0070 (7)
C3	0.0760 (12)	0.0395 (8)	0.0797 (12)	0.0054 (8)	0.0213 (9)	−0.0107 (8)
C4	0.0801 (12)	0.0566 (10)	0.0543 (9)	−0.0021 (9)	0.0172 (8)	−0.0203 (8)
C5	0.0625 (9)	0.0468 (8)	0.0391 (7)	0.0006 (7)	0.0110 (6)	−0.0035 (6)
C6	0.0414 (6)	0.0348 (6)	0.0368 (6)	−0.0027 (5)	0.0134 (5)	−0.0009 (5)
C7	0.0393 (6)	0.0353 (6)	0.0294 (5)	−0.0010 (5)	0.0113 (4)	0.0006 (4)
C8	0.0462 (7)	0.0358 (6)	0.0366 (6)	−0.0001 (5)	0.0140 (5)	0.0002 (5)
C9	0.0375 (6)	0.0348 (6)	0.0306 (5)	0.0003 (4)	0.0115 (4)	0.0012 (4)
C10	0.0411 (6)	0.0366 (6)	0.0321 (5)	0.0030 (5)	0.0139 (5)	0.0021 (4)
C11	0.0695 (9)	0.0288 (6)	0.0409 (6)	−0.0015 (6)	0.0231 (6)	−0.0010 (5)
C12	0.0381 (6)	0.0336 (5)	0.0307 (5)	0.0010 (4)	0.0121 (4)	0.0003 (4)

### Geometric parameters (Å, °)

**Table:**

S1—C12	1.6735 (13)	C2—C3	1.372 (3)
N1—C7	1.3358 (15)	C2—H2A	0.9300
N1—N2	1.3516 (16)	C3—C4	1.380 (3)
N2—C8	1.3283 (17)	C3—H3A	0.9300
N2—H1N2	0.84 (2)	C4—C5	1.390 (2)
N3—C10	1.2731 (17)	C4—H4A	0.9300
N3—N4	1.3950 (14)	C5—C6	1.3880 (19)
N4—C11	1.3693 (16)	C5—H5A	0.9300
N4—C12	1.3830 (15)	C6—C7	1.4744 (17)
N5—C11	1.2905 (18)	C7—C9	1.4160 (17)
N5—N6	1.3700 (16)	C8—C9	1.3862 (17)
N6—C12	1.3433 (16)	C8—H8A	0.9300
N6—H1N6	0.90 (2)	C9—C10	1.4460 (16)
C1—C2	1.382 (2)	C10—H10A	0.9300
C1—C6	1.3916 (19)	C11—H11A	0.9300
C1—H1A	0.9300		
			
C7—N1—N2	105.45 (10)	C6—C5—C4	119.74 (15)
C8—N2—N1	112.74 (11)	C6—C5—H5A	120.1
C8—N2—H1N2	128.2 (13)	C4—C5—H5A	120.1
N1—N2—H1N2	119.0 (13)	C5—C6—C1	118.92 (13)
C10—N3—N4	117.80 (11)	C5—C6—C7	121.48 (12)
C11—N4—C12	107.64 (10)	C1—C6—C7	119.60 (12)
C11—N4—N3	118.92 (10)	N1—C7—C9	110.02 (11)
C12—N4—N3	133.20 (10)	N1—C7—C6	120.03 (11)
C11—N5—N6	103.26 (11)	C9—C7—C6	129.93 (11)
C12—N6—N5	114.42 (11)	N2—C8—C9	106.89 (11)
C12—N6—H1N6	125.9 (14)	N2—C8—H8A	126.6
N5—N6—H1N6	119.6 (14)	C9—C8—H8A	126.6
C2—C1—C6	120.79 (15)	C8—C9—C7	104.90 (11)
C2—C1—H1A	119.6	C8—C9—C10	127.30 (12)
C6—C1—H1A	119.6	C7—C9—C10	127.52 (11)
C3—C2—C1	120.07 (16)	N3—C10—C9	119.17 (12)
C3—C2—H2A	120.0	N3—C10—H10A	120.4
C1—C2—H2A	120.0	C9—C10—H10A	120.4
C2—C3—C4	119.84 (15)	N5—C11—N4	112.28 (12)
C2—C3—H3A	120.1	N5—C11—H11A	123.9
C4—C3—H3A	120.1	N4—C11—H11A	123.9
C3—C4—C5	120.62 (16)	N6—C12—N4	102.40 (10)
C3—C4—H4A	119.7	N6—C12—S1	126.82 (9)
C5—C4—H4A	119.7	N4—C12—S1	130.77 (9)
			
C7—N1—N2—C8	0.32 (16)	N2—C8—C9—C7	−0.12 (15)
C10—N3—N4—C11	164.74 (13)	N2—C8—C9—C10	174.12 (13)
C10—N3—N4—C12	−21.8 (2)	N1—C7—C9—C8	0.33 (15)
C11—N5—N6—C12	−0.19 (18)	C6—C7—C9—C8	178.66 (13)
C6—C1—C2—C3	0.0 (3)	N1—C7—C9—C10	−173.91 (13)
C1—C2—C3—C4	0.8 (3)	C6—C7—C9—C10	4.4 (2)
C2—C3—C4—C5	−0.4 (3)	N4—N3—C10—C9	−178.11 (11)
C3—C4—C5—C6	−0.8 (3)	C8—C9—C10—N3	2.6 (2)
C4—C5—C6—C1	1.6 (2)	C7—C9—C10—N3	175.63 (13)
C4—C5—C6—C7	−178.29 (14)	N6—N5—C11—N4	0.09 (18)
C2—C1—C6—C5	−1.2 (2)	C12—N4—C11—N5	0.03 (18)
C2—C1—C6—C7	178.68 (15)	N3—N4—C11—N5	175.07 (12)
N2—N1—C7—C9	−0.39 (15)	N5—N6—C12—N4	0.20 (16)
N2—N1—C7—C6	−178.91 (12)	N5—N6—C12—S1	−179.73 (11)
C5—C6—C7—N1	−143.18 (14)	C11—N4—C12—N6	−0.14 (15)
C1—C6—C7—N1	36.93 (19)	N3—N4—C12—N6	−174.18 (13)
C5—C6—C7—C9	38.6 (2)	C11—N4—C12—S1	179.79 (12)
C1—C6—C7—C9	−141.26 (15)	N3—N4—C12—S1	5.8 (2)
N1—N2—C8—C9	−0.12 (16)		

### Hydrogen-bond geometry (Å, °)

**Table:**

*D*—H···*A*	*D*—H	H···*A*	*D*···*A*	*D*—H···*A*
N2—H1N2···S1^i^	0.84 (2)	2.59 (2)	3.3593 (15)	153.6 (17)
N6—H1N6···N1^ii^	0.90 (2)	1.91 (2)	2.7884 (15)	168.0 (2)
C10—H10A···S1	0.93	2.50	3.2183 (13)	134

Symmetry codes: (i) *x*+1, −*y*+3/2, *z*−1/2; (ii) *x*−1, −*y*+3/2, *z*+1/2.
